# LPAR1 regulates the development of intratumoral heterogeneity in ovarian serous cystadenocarcinoma by activating the PI3K/AKT signaling pathway

**DOI:** 10.1186/s12935-019-0920-0

**Published:** 2019-07-29

**Authors:** Ran Cui, Guangming Cao, Huimin Bai, Zhenyu Zhang

**Affiliations:** Department of Obstetrics and Gynecology, Beijing Chao-Yang Hospital, Capital Medical University, No.8, North Road of Workers Stadium, Chaoyang District, Beijing, 100020 China

**Keywords:** Ovarian serous cystadenocarcinoma, Intratumoral heterogeneity, LPAR1, PI3K/AKT pathway, Single-cell subclones

## Abstract

**Background:**

To explore the role of lysophosphatidic acid receptor 1 (LPAR1) and its correlation with the PI3K/AKT pathway in the development of intratumoral heterogeneity (ITH) in human ovarian serous cystadenocarcinoma (OSC).

**Methods:**

Immunohistochemical staining was performed to detect LPAR1 expression in matched primary and recurrent lesions from the same patients. Cell models of ITH were established using the limiting dilution methodology and Transwell invasion/migration assays. LPAR1 expression in the ITH cell models was silenced or upregulated with lentiviral particles, and the biological characteristics were evaluated using various in vitro and in vivo assessments of cell function. The levels of phosphorylated PI3K/AKT (p-PI3K/p-AKT) in LPAR1 knockdown and LPAR1-overexpressing cells were detected.

**Results:**

The H-scores for LPAR1 staining in the lymphatic metastatic and recurrent lesions were noticeably higher than in the primary tumor lesions from the same patients (P = 0.024/0.031). High LPAR1 expression was associated with worse progression-free survival and overall survival (P = 0.017/0.039). Biological functions in vitro, including invasion, migration, and proliferation, and tumor formation in vivo were decreased in the LPAR1-silenced cells (all P < 0.05). These cellular functions were significantly increased in the LPAR1-overexpressing cells in vitro and in vivo (all P < 0.05). The levels of p-PI3K and p-AKT were significantly decreased in the LPAR1 knockdown cells and significantly increased in the LPAR1-overexpressing cells (all P < 0.05).

**Conclusions:**

Higher levels of the LPAR1 protein were associated with a poor prognosis. LPAR1 plays essential roles in the invasion, migration, and proliferation of heterogeneous subsets of OSC cell lines and the development of ITH of OSC, possibly by modulating the activity of the PI3K/AKT signaling pathway.

**Electronic supplementary material:**

The online version of this article (10.1186/s12935-019-0920-0) contains supplementary material, which is available to authorized users.

## Background

Ovarian cancer is the most lethal gynecological carcinoma, and limited improvements in the survival of patients with advanced-stage ovarian cancer have been achieved over the last few decades [1]. Tumor metastasis, drug resistance and recurrence are the major causes of a poor prognosis in patients [2, 3]. Within a single tumor, cancer cells display various heterogeneous features, including different biological characteristics, gene expression levels, and differentiation statuses; this phenomenon is referred to as intratumoral heterogeneity (ITH) [4–6]. ITH appears to be related to tumor metastasis, therapeutic resistance and recurrence, which lead to treatment failure in many human malignant tumors [7–11].

In our previous study, two pairs of single-cell subclones (named as A-H/A-L and S-H/S-L) with distinct invasive/migratory capacities were isolated and established from the same human ovarian serous cystadenocarcinoma (OSC) cell lines (SKOV3 and A2780) using the limiting dilution methodology [11, 12]; therefore, these cell lines have the same genetic backgrounds [11]. Compared with the A-L/S-L cells, the A-H/S-H cells exhibited significantly more aggressive phenotypes with respect to their biological functions in vitro and tumor formation in vivo. These two pairs of single-cell subclones are considered ideal cell models of ITH in human OSC. RNA-Seq and bioinformatics analyses revealed that the expression of components of the PI3K/AKT pathway, including lysophosphatidic acid receptor 1 (LPAR1), was significantly increased in the A-H/S-H cells. LPAR1 was the first identified high-affinity receptor for lysophosphatidic acid (LPA) and belongs to the G protein-coupled receptor (GPCR) superfamily [13]. LPAR1 binds to and activates three types of G proteins (G_αi/o_ o, 13G_αq/11_, and G_α12/13_), which convey signals through downstream molecules and signaling pathways, including the PI3K/AKT pathway [13–15]. LPAR1 might play a vital role in various benign and malignant diseases by activating the PI3K/AKT pathway [16–18]. Based on the results of our previous study and a literature review, we hypothesized that LPAR1 might be involved in the development of ITH in human OSC by activating the PI3K/AKT pathway. This study aimed to explore the role of LPAR1 and its correlation with the PI3K/AKT pathway in the development of ITH.

## Materials and methods

The principal materials and instruments used in the present study are listed in Additional file 1: Table S1.

### Clinical data and tissue specimens

After obtaining approval from the Beijing Chao-Yang Hospital Ethics Committee, patients with OSC who were diagnosed with high-grade serous carcinoma (HGSC) based on pathological examination and underwent cytoreductive surgery and standard platinum/paclitaxel chemotherapy as the initial treatment at Beijing Chao-Yang Hospital between April 2006 and May 2015 were included in the present study. Additionally, matched paraffin-embedded tissues from these patients were collected from the pathology centre. The tissue specimens, including primary tumor lesions, abdominal disseminated lesions at presentation, lymphatic metastatic lesions at presentation and recurrent lesions, were all obtained from the same patients if possible. The four types of OSC tissue specimens from the same patients were matched for the detection of the heterogeneous expression of LPAR1 and an exploration of ITH in OSC. The clinical and pathological data for these patients were retrospectively collected and reviewed. The stages were reassessed based on the 2014 International Federation of Gynecology and Obstetrics (FIGO) system [19]. The progression-free survival (PFS) was calculated from the date of surgery to the date of recurrence; women who were disease-free at the time of their last visit were censored. The overall survival (OS) time was calculated from the date of surgery to the date of patient death from the disease, and patients who died from other conditions and survivors at the time of their last visit were censored.

### Tissue microarray (TMA)

TMAs of matched primary tumor lesions, abdominal disseminated lesions, lymphatic metastatic lesions and recurrent lesions obtained from the same patients with OSC were constructed. The pathology slides were re-examined by two independent gynecological pathologists to confirm the diagnosis of OSC, and the accurate locations of the tumors were marked on the paraffin-embedded tissue samples. Round tissue samples with a diameter of 1 millimetre were obtained from the tumor located in the donor block using a manual tissue array instrument (TMArrayer), and transferred into the TMA block (10 × 12 arrays). Sequential sections were cut from the paraffin-embedded TMA blocks at a thickness 4 μm and placed on blank slides.

### Immunohistochemical (IHC) staining

IHC staining was performed as previously described [20]. After baking the tissue sections at 70 °C for 60 min, the sections were deparaffinized, rehydrated, treated with 3% hydrogen peroxide to block endogenous peroxidase activity, and subjected to antigen retrieval (citrate buffer solution). After blocking with 10% goat serum at room temperature for 60 min, tissue sections were incubated with the primary antibody (anti-LPAR1; 1:100) at 4 °C overnight. After an incubation with a horseradish peroxidase-conjugated goat anti-rabbit antibody for 60 min, tissue sections were subjected to diaminobenzidine staining for color development. Subsequently, sections were subjected to hematoxylin counterstaining and dehydration, and then sealed with neutral resin.

### Evaluation of the IHC staining of the TMA

A digital pathological section scanner (Pannoramic MIDI/P250) was utilized to capture images of the TMA slides, and the images were displayed at 1 to 400× magnification using Pannoramic Viewer 1.15.4 software. All images were independently evaluated by two independent gynecological pathologists who were blinded to the clinical data. A histochemistry score (H-score) based on a combination of the percentage of stained cells and staining intensity was calculated for the semiquantitative analysis. H-score = ∑(percentage [0–100%] × intensity [1–3]) = (percentage of cells with weak intensity × 1) + (percentage of cells with moderate intensity × 2) + (percentage of cells with strong intensity × 3) [21]. The differences in LPAR1 expression among the four types of OSC lesions from the same patients were compared. In addition, the relationship between LPAR1 expression and the patients’ prognosis was analyzed. A poor prognosis was defined as PFS for less than 12 months or OS for less than 36 months.

### Cell culture and establishment of cell models of ITH

Based on our previous study, two pairs of single-cell subclones with distinct invasive/migratory capacities (named A-H/A-L/S-H/S-L) derived from SKOV3 and A2780 cells may develop heterogeneous changes due to repeated passaging [11]. Thus, single-cell subclones were reisolated from A-H/S-H and A-L/S-L cells using the limiting dilution methodology as described in detail in our previous study. The A-H/S-H and A-L/S-L cells were cultured in RPMI-1640 supplemented with 10% fetal bovine serum (FBS) and antibiotics (100 U/ml penicillin and 0.1 mg/ml streptomycin) at 37 °C in a humidified incubator with a 5% CO_2_ atmosphere. During the logarithmic growth phase, A-H, A-L, S-H, and S-L cells were diluted to 10 cells/ml and inoculated in 96-well plates with 100 μl per well. At 4 to 6 h after inoculation, single adherent cells were observed under a microscope, and these cells were marked for continuous daily observation. Once the cells had proliferated to a certain density, single-cell subclones were successively transferred to 48-, 24-, and 6-well plates and then culture dishes; subsequently, the cells were conventionally cultured. Transwell invasion/migration assays were performed to select single-cell subclones with distinct invasive/migratory capacities, which were used as cell models of ITH in vitro. For further comparative analyses, single-cell subclones from similar generations within the 20th generation were used to avoid heterogeneous changes due to repeated passaging [11].

### Transwell invasion/migration assays

The Transwell invasion/migration assays were performed using previously described method [22]. The Transwell chambers were either coated (invasion assay) or uncoated (migration assay) with 40 μl of Matrigel diluted 1:10 in serum-free RPMI-1640. Cells suspended in 200 µl of RPMI-1640 supplemented with 0.1% FBS were seeded in the upper portions of the chambers at a density of 8 × 10^4^ cells/well (invasion assay) or 4 × 10^4^ cells/well (migration assay). RPMI-1640 (600 μl) supplemented with 10% FBS, which was regarded as a chemotactic factor, was added to the lower portions of the chambers. After 24–36 h of incubation at 37 °C, cells were fixed with paraformaldehyde (4%) and stained with crystal violet. The noninvading/nonmigrating cells on the upper surface of the filter were carefully and completely removed with cotton swabs, and then the Transwell insert was observed and photographed under a microscope. Image-Pro Plus software was used to calculate the number of stained cells on the lower surface of the membrane. Each assay was performed in triplicate.

### Cell proliferation assay with Counting Kit-8 (CCK-8)

Cells (3 × 10^3^/well, 6 wells) were seeded in 96-well plates in quadruplicate. The CCK-8 reagent was added to four 96-well plates after incubations for 4, 24, 48, or 72 h. After 2 h of incubation with the CCK-8 reagent, the absorbance was measured at 450 nm using an enzyme-linked immunosorbent assay plate reader. The assay was performed in triplicate.

### Western blotting

Western blotting was performed as previously described [11]. The total protein was extracted from cells in the logarithmic growth phase using radioimmunoprecipitation assay lysis buffer containing protease and phosphatase inhibitors. Proteins (30 μg per group) were separated using 10% sodium dodecyl sulfate-polyacrylamide gel electrophoresis and transferred to a nitrocellulose membrane. After blocking with 5% nonfat milk at room temperature for 2 h, the nitrocellulose membrane was incubated with the primary antibody overnight at 4 °C. The primary antibodies are listed in Additional file 2: Table S2. After an incubation with a horseradish peroxidase-conjugated secondary IgG antibody (1:3000) at room temperature for 2 h, the nitrocellulose membrane was visualized using electrochemiluminescence (Bio-Rad GelDoc EZ), and the images were semi-quantitatively analyzed using ImageJ software. Each assay was performed in triplicate.

#### Quantitative real-time polymerase chain reaction (qRT-PCR)

qRT-PCR was performed as previously described [11]. Total RNA was extracted from cells in the logarithmic growth phase using TRIzol reagent. The RNA concentration was determined using a NanoDrop ND-2000 spectrophotometer. Then, cDNAs were synthesized using a PrimeScript RT Reagent Kit with gDNA Eraser. qRT-PCR was performed using an ABI Prism 7500 RT-PCR system. The relative mRNA levels were normalized to levels of the endogenous control (GAPDH) and calculated using the 2^−ΔCT^ comparison method. The following primers were used: LPAR1 forward, 5′-CTTTGCTGGGTTGGCCTACTT-3′, and reverse, 5′-GCCATGTGCTAACAGTCAGTCT-3′, and GAPDH forward, 5′-AAGGTCATCCCTGAGCTGAAC-3′, and reverse, 5′-ACGCCTGCTTCACCACCTTCT-3′. Each assay was performed in triplicate.

### Production and transduction of lentiviral particles

Three specific short hairpin RNAs (shRNAs) targeting the LPAR1 gene, as well as a scrambled shRNA (negative control, NC), were designed and synthesized (Additional file 3: Table S3). Separate fragments containing different shRNAs targeting LPAR1 and the scrambled shRNA sequence were cloned into the GV248 plasmid. Subsequently, the GV248 plasmid and other packaging plasmids were cotransfected into HEK293T cells using a Lipofectamine 2000, and the viral particles were collected 48 h after transfection. After collecting cells infected with viral particles and extracting proteins and RNAs, the viral particles containing the most effective shRNA sequence (CTATGAGAAATTCTTCCTT) were selected based on the results of Western blotting and qRT-PCR in the preliminary experiment, which were named Lv-LPAR1-shRNA. The viral particles containing the scrambled shRNA were named Lv-LPAR1-shRNA-NC.

The LPAR1 cDNA was ligated into a lentiviral vector, i.e., the GV492 plasmid, to amplify the LPAR1 gene. The LPAR1 gene was amplified using the forward primer gGGATCCCGCCACCATGGCTGCCATC and the reverse primer gACCGGTAACCACAGAGTGG. The PCR products were cleaved using BamH I and Ape I and ligated into the lentiviral vector (the GV492 plasmid). The GV492-LPAR1 plasmid and other packaging plasmids were cotransfected into HEK293T cells using the same method described above, and the viral particles were named Lv-LPAR1. Additionally, a GV492 plasmid vector lacking the LPAR1 insert was also transfected into HEK293T cells to obtain a control virus (Lv-LPAR1-NC).

The cells were seeded in 24-well plates at a density of 5 × 10^4^ cells per well. After incubating the cells for 24 h, the lentiviral particles were diluted in enhanced infection solution at different multiplicities of infection, and polybrene (5 μg/ml) was added to the cells. The medium (RPMI-1640 supplemented with 10% FBS) was changed after 8–12 h. At 72 h after infection, cells that had been infected with lentiviral vectors encoding enhanced green fluorescent protein (eGFP) were observed under a fluorescence microscope to detect the transduction efficiency and eGFP expression. Cells with 80% infection efficiency and good proliferation were selected and expanded in the presence of puromycin. The efficiency of the silencing and amplification of LPAR1 was confirmed using Western blotting and qRT-PCR.

### Xenograft experiments

All procedures performed in animal studies were approved by the Animal Research Ethics Committee of Capital Medical University. Seventy-two female nude mice (female BALB/c, 4 weeks of age) were randomly divided into 12 groups (6 mice per group). After harvesting and resuspending tumor cells in phosphate-buffered saline, 3 × 10^6^ cells were subcutaneously injected into the right flank of each nude mouse. The tumor width and length were measured every 5 days with digital calipers. The tumor volumes were calculated using the formula (width)^2^ × length/2. The nude mice were sacrificed via CO_2_ inhalation after 30 to 60 days of observation, depending on the tumor growth rate. The tumors were isolated, fixed with 10% formalin, and embedded in paraffin for further pathological analyses.

### Statistical analysis

Statistical analyses were performed using the SPSS 22.0 statistical package. The quantitative data are presented as mean ± SD and were analyzed using ANOVA or two-tailed Student’s t-tests. The Wilcoxon signed rank test was used to compare the differences in LPAR1 expression between HGSC lesions obtained from two different sites in the same patients. The two-sample rank sum test was used to analyze the relationship between LPAR1 expression and the prognosis. A two-sided P-value < 0.05 was considered statistically significant.

## Results

### Heterogeneous expression of LPAR1 in human OSC tissues

**Fig. 1:**
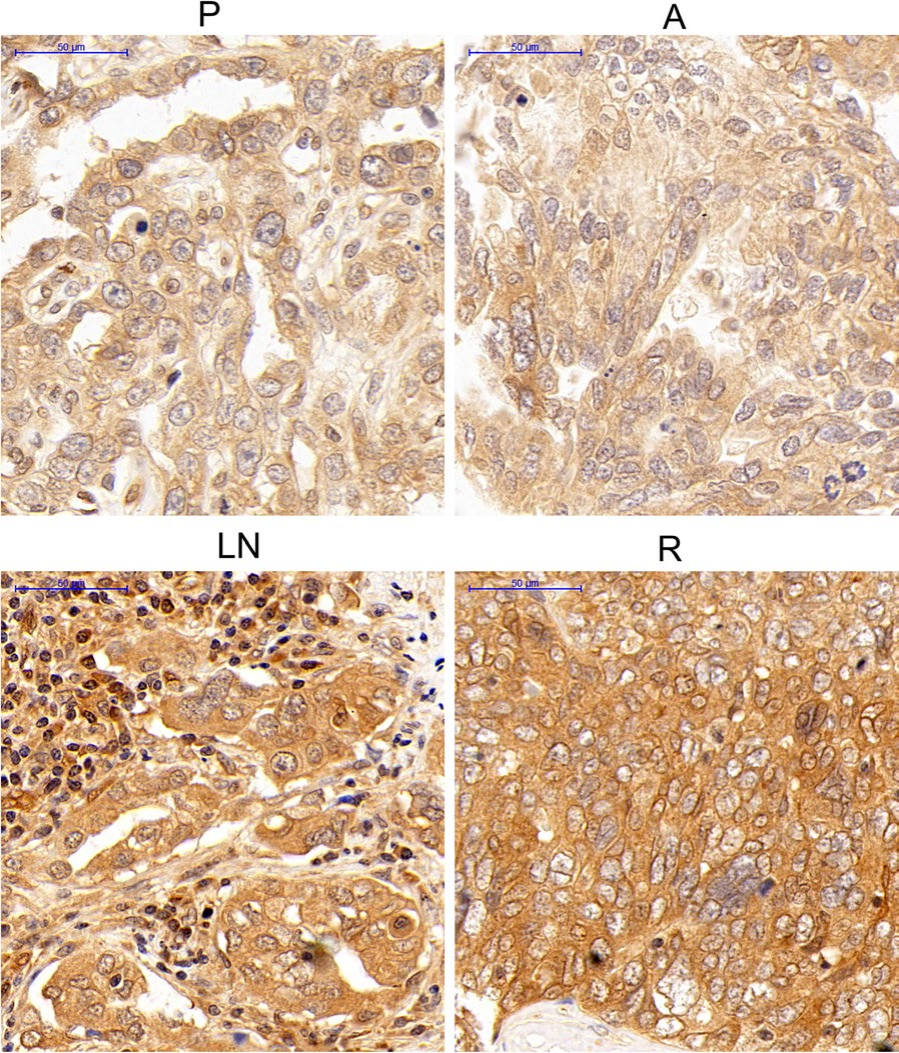
IHC staining for the LPAR1 protein in four types of matched lesions from a patient with OSC. In the image of IHC staining from the same patient, increased LPAR1 staining was observed in the recurrent lesions and lymphatic metastatic lesions compared with the primary tumor lesions. However, no differences in LPAR1 expression were observed between the primary tumor lesions and abdominal disseminated lesions. Original magnification: ×400. *P* primary tumor samples, *A* abdominal disseminated samples, *LN* lymph node metastases samples, *R* recurrent samples

Seventy-four patients with OSC were included in the present study, and the clinicopathological characteristics of these patients are presented in Table 1. The mean age at diagnosis was 53.41 ± 9.29 (range 34–76) years. All included patients were diagnosed with HGSC. Based on the FIGO 2014 staging system, 10, 6, 52, and 6 patients had stage I, stage II, stage III, and stage IV tumors, respectively. The TMAs included 166 OSC tissue specimens, including primary tumor lesions (74), abdominal disseminated lesions (52), lymphatic metastatic lesions (28) and recurrent lesions (12). The recurrent lesions included pelvic masses, lymph nodes, rectal tissues, and colon tissues, which were obtained through biopsy or open-abdominal surgery. The clinicopathological characteristics of these patients with recurrent OSC are presented in Table 2. The numbers of primary tumor lesions matching the abdominal disseminated lesions, lymphatic metastatic lesions and recurrent lesions from the same patients with OSC were 52, 28, and 12, respectively. IHC staining was performed to detect LPAR1 expression in the matched specimens from the same patients. Compared with the primary tumor lesions, increased LPAR1 staining was observed in the recurrent and lymphatic metastatic lesions from the same patients (Fig. 1). The H-scores for LPAR1 staining in the lymphatic metastatic lesions and recurrent lesions were noticeably higher than the primary tumor lesions (159.08 ± 27.23 vs 145.69 ± 29.45, P = 0.024; 165.25 ± 21.49 vs 145.69 ± 29.45, P = 0.031, respectively) (Table 3). However, no differences in the H-scores for LPAR1 staining were not observed between the primary tumor lesions and abdominal disseminated lesions (145.69 ± 29.45 vs 147.79 ± 30.64, P = 0.152). In addition, the H-score for LPAR1 staining in the primary tumor lesions was significantly higher in the patients with a PFS of less than 12 months or OS of less than 36 months (P = 0.017/0.039), indicating that higher levels of the LPAR1 protein were associated with a worse prognosis (Table 4).

**Table 1:** Clinicopathological characteristics of the 74 patients with HGSC

	Mean or number	Range or percentage
Age at diagnosis (years)	53.41 ± 9.29	34–76
Menopausal status		
Pre-menopausal	23	31.1%
Post-menopausal	51	68.9%
Family history of cancer		
Yes	6	8.1%
No	68	91.9%
Preoperative CA125 levels (IU/ml)	2005.42 ± 3137.71	15–17,980
FIGO stage		
Stage I	10	13.5%
Stage II	6	8.1%
Stage III	52	70.3%
Stage IV	6	8.1%
Lymphatic metastasis		
Yes	28	37.8%
No	46	62.2%
Cytoreductive surgery		
Residual tumor ≤ 1 cm	53	71.6%
Residual tumor > 1 cm	22	29.7%
Current status		
NED	25	33.8%
AWD	19	25.7%
DOD	30	40.5%

*HGSC* high-grade sercous carcinoma, *FIGO* International Federation of Gynecology and Obstetrics, *NED* no evidence of disease, *AWD* alive with disease, *DOD* die of disease

**Table 2:** The clinicopathological characteristics of these patients with recurrent HGSC

Patient	FIGO stage	Recurrent site	Recurrence interval (months)	Recurrent lesions obtained for IHC	Method used to obtain specimens	Current status
1	IVB	Brain	10	Pelvic masses	Open-abdominal surgery	DOD
2	IIIB	Pelvic cavity	18	Rectum	Open-abdominal surgery	DOD
3	IIIC	Pelvic/abdominal cavity	29	Transverse colon	Open-abdominal surgery	DOD
4	IIIC	Pelvic cavity	11	Pelvic masses	Open-abdominal surgery	DOD
5	IIIB	Pelvic cavity	45	Pelvic masses	Open-abdominal surgery	DOD
6	IIIC	Pelvic cavity	21	Pelvic masses	Open-abdominal surgery	DOD
7	IIIB	Lymph nodes	18	Inguinal lymph nodes	Biopsy	AWD
8	IIIC	Lymph nodes	20	Supraclavicular lymph nodes	Biopsy	DOD
9	IIA	Pelvic cavity	32	Pelvic masses	Open-abdominal surgery	AWD
10	IVB	Lymph nodes	6	Inguinal lymph nodes	Biopsy	DOD
11	IIIB	Pelvic/abdominal cavity	25	Right colon	Open-abdominal surgery	AWD
12	IIIB	Pelvic/abdominal cavity	47	Transverse colon	Open-abdominal surgery	AWD

*HGSC* high-grade sercous carcinoma, *FIGO* International Federation of Gynecology and Obstetrics, *IHC* immunohistochemistry, *DOD* die of disease, *AWD* alive with disease

**Table 3:** The H-scores for LPAR1 staining in four types of HGSC tissue specimens

	Number	LPAR1 H-score/P^a^ value
Primary samples	74	145.69 ± 29.45
Abdominal disseminated samples	52	147.79 ± 30.64
Lymphatic metastatic samples	28	159.08 ± 27.23
Recurrent samples	12	165.25 ± 21.49
Primary samples vs disseminated samples	52	0.152
Primary samples vs Lymphatic metastatic samples	28	*0.024*
Primary samples vs Recurrent samples	12	*0.031*

Italic values indicate statistical significance (P < 0.05)

*HGSC* high-grade sercous carcinoma, *LPAR1* lysophosphatidic acid receptor 1

^a^Wilcoxon’s signed rank test

**Table 4:** The relationship between LPAR1 expression and prognosis

	Primary samples n = 74		P value	Primary samples n = 74		P value
	PFS < 12 months n = 16	PFS ≥ 12 months n = 58		OS < 36 months n = 38	OS ≥ 36 months n = 36	
LPAR1	143.76 ± 18.93	136.65 ± 23.34	*0.017*^a^	148.20 ± 19.62	137.43 ± 26.67	*0.039*^a^

Italic values indicate statistical significance (P < 0.05)

*LPAR1* lysophosphatidic acid receptor 1, *PFS* progression-free survival, *OS* overall survival

^a^Two samples rank sum test

### Establishment of cell models of ITH in vitro

**Fig. 2:**
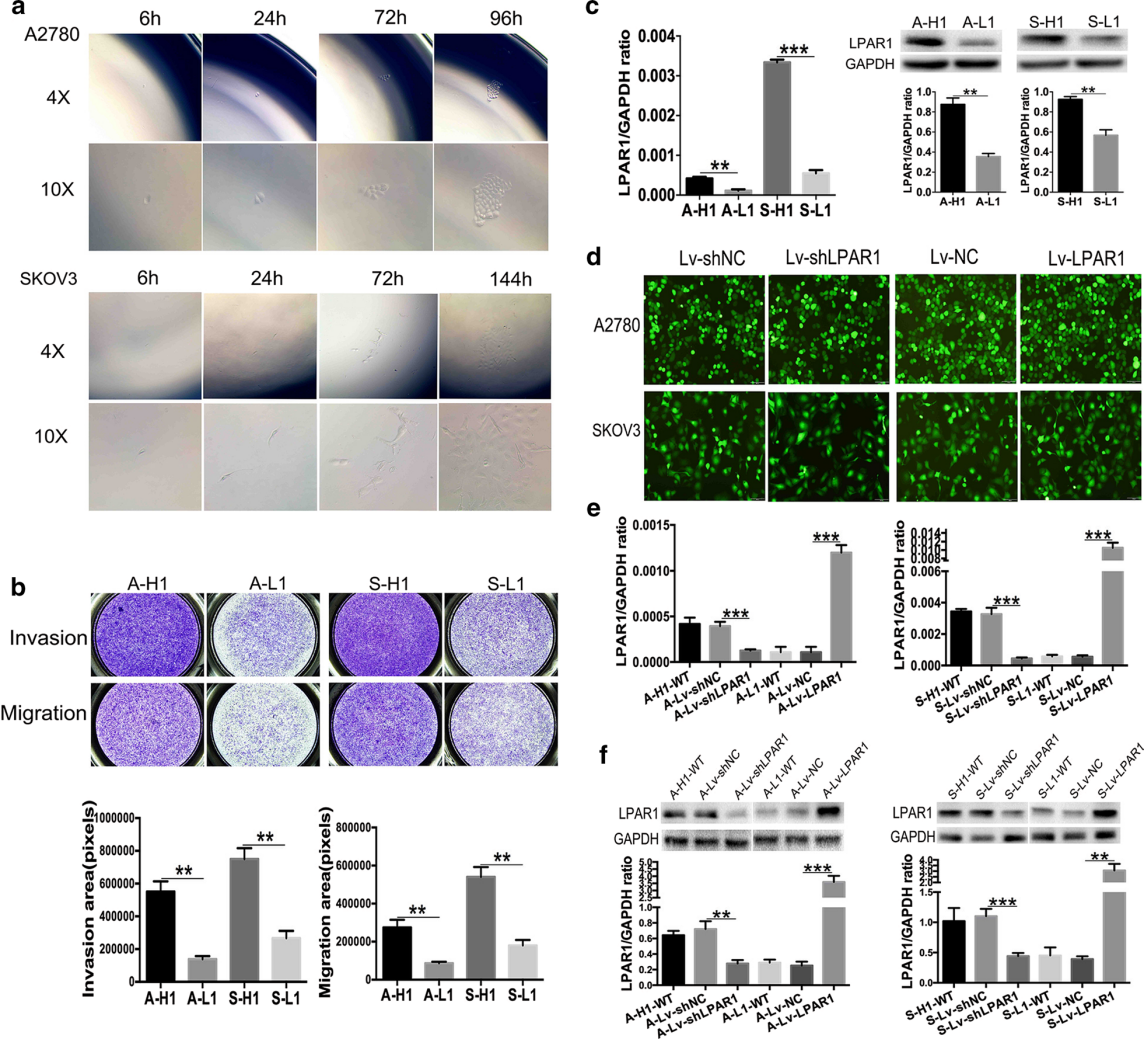
Establishment of cell models of ITH and stable LPAR1 knockdown and LPAR1-overexpressing cells. **a** Isolation and amplification of single-cell subclones (A2780 and SKOV3 cells) using the limited dilution methodology. At 4 to 6 h after inoculation, single adherent cells were observed; and over time, the cell number continued to increase. **b** Two pairs of single-cell subclones with distinct invasive/migratory capacities were selected using Transwell invasive/migratory assays; these subclones were designated A-H1 (A2780 high), A-L1 (A2780 low), S-H1 (SKOV3 high), and S-L1 (SKOV3 low). Significantly greater invasion/migration were observed for the A-H1/S-H1 cells than for the A-L1/S-L1 cells (invasion: A2780: 550,839 ± 62,590 vs 138,417 ± 19,075 P = 0.003; SKOV3: 750,693 ± 65,709 vs 267,164 ± 43,846, P = 0.004) (migration: A2780: 274,674 ± 40,009 vs 87,295 ± 7186, P = 0.010; SKOV3: 540,902 ± 51,325 vs 180,497 ± 28,749, P = 0.004). **c** qRT-PCR (**a**) and Western blotting (**b**) consistently confirmed significantly higher levels of the LPAR1 mRNA and protein in A-H1/S-H1 cells than in the A-L1/S-L1 cells (qRT-PCR: P = 0.004/< 0.001; Western blotting: P = 0.002/0.004). **d** A2780 and SKOV3 cells infected with plasmid a containing eGFP were observed under a fluorescence microscope. Lv-shLPAR1 and Lv-LPAR1 represented LPAR1 knockdown and LPAR1-overexpressing cells, respectively. Lv-shNC and Lv-NC were administered to the control groups. Scale bars indicate 100 μm. qRT-PCR (**e**) and Western blotting (**f**) consistently indicated the significant and stable downregulation of the LPAR1 mRNA and protein in the LPAR1 knockdown cells and significant and stable upregulation in the LPAR1-overexpressing cells (qRT-PCR: both P < 0.001 [A2780]; both P < 0.001 [SKOV3]) (Western blotting: P = 0.002/< 0.001 [A2780]; P < 0.001/= 0.001 [SKOV3]). In addition, no differences were observed between the control groups and the corresponding wild-type (WT) groups (all P > 0.05)

Two pairs of single-cell subclones with distinct invasive/migratory capacities established in our previous study (named A-H/A-L/S-H/S-L) were seeded in two 96-well plates using the limiting dilution methodology. At 4 to 6 h after isolation and inoculation, single adherent cells were observed, and over time, the cell number continued to increase (Fig. 2a). The single-cell subclones were transferred from 96-well plates to 48-, 24-, and 6-well plates and, ultimately, 25-cm square culture flasks within 6 to 10 weeks. Sixteen A-H, 13 A-L, 15 S-H, and 12 S-L single-cell subclones were obtained. The single-cell subclones exhibiting the highest and lowest invasive/migratory capacities were selected based on the results from Transwell invasion/migration assays (Fig. 2b); these subclones were renamed A-H1 (A2780 high), A-L1 (A2780 low), S-H1 (SKOV3 high), and S-L1 (SKOV3 low). The Transwell invasion/migration assays revealed significantly greater invasive/migratory capacities of A-H1 cells than A-L1 cells (invasion: 550,839 ± 62590 vs 138,417 ± 19,075, P = 0.003; migration: 274,674 ± 40,009 vs 87,295 ± 7186, P = 0.010). Similarly, the S-H1 cells and S-L1 cells exhibited the most distinct differences in invasion/migration (invasion: 750,693 ± 65,709 vs 267,164 ± 43,846, P = 0.004; migration: 540,902 ± 51,325 vs 180,497 ± 28,749, P = 0.004). The levels of the LPAR1 protein and mRNA were detected using Western blotting and qRT-PCR, respectively. The statistical analyses consistently revealed significantly increased levels of the LPAR1 mRNA and protein in the A-H1/S-H1 cells than in the A-L1/S-L1 cells (Fig. 2c) (qRT-PCR: P = 0.004/< 0.001; Western blotting: P = 0.002/0.004).

### Establishment of stable LPAR1 knockdown and LPAR1-overexpressing cells

Stable LPAR1 knockdown and LPAR1-overexpressing cell lines were established by transducing the cells with lentiviral particles. The A-H1 and S-H1 cells infected with Lv-LPAR1-shRNA were stable LPAR1 knockdown cells, which were named A-Lv-shLPAR1 and S-Lv-shLPAR1 cells, respectively. The A-H1 and S-H1 cells infected with Lv-LPAR1-shRNA-NC were named A-Lv-shNC and S-Lv-shNC cells, respectively, and were considered the control groups for the knockdown groups. Similarly, the A-L1 and S-L1 cells infected with Lv-LPAR1 were LPAR1-overexpressing cells, which were named A-Lv-LPAR1 and S-Lv-LPAR1 cells, respectively. The A-L1 and S-L1 cells infected with Lv-LPAR1-NC were named A-Lv-NC and S-Lv-NC, respectively, and were considered the control groups for the overexpressing groups. At 72 h after infection, cells infected with plasmids containing eGFP were observed under a fluorescence microscope (Fig. 2d). qRT-PCR and Western blotting were conducted to detect LPAR1 levels. The statistical analyses consistently indicated significant and stable downregulation of the LPAR1 mRNA and protein in the LPAR1 knockdown cells and significant and stable upregulation of the LPAR1 mRNA and protein in the LPAR1-overexpressing cells (Fig. 2e, f) (all P < 0.01). In addition, the control groups and the corresponding wild-type groups exhibited similar levels of the LPAR1 protein and mRNA (all P > 0.05).

### Role of LPAR1 in biological functions in vitro

**Fig. 3:**
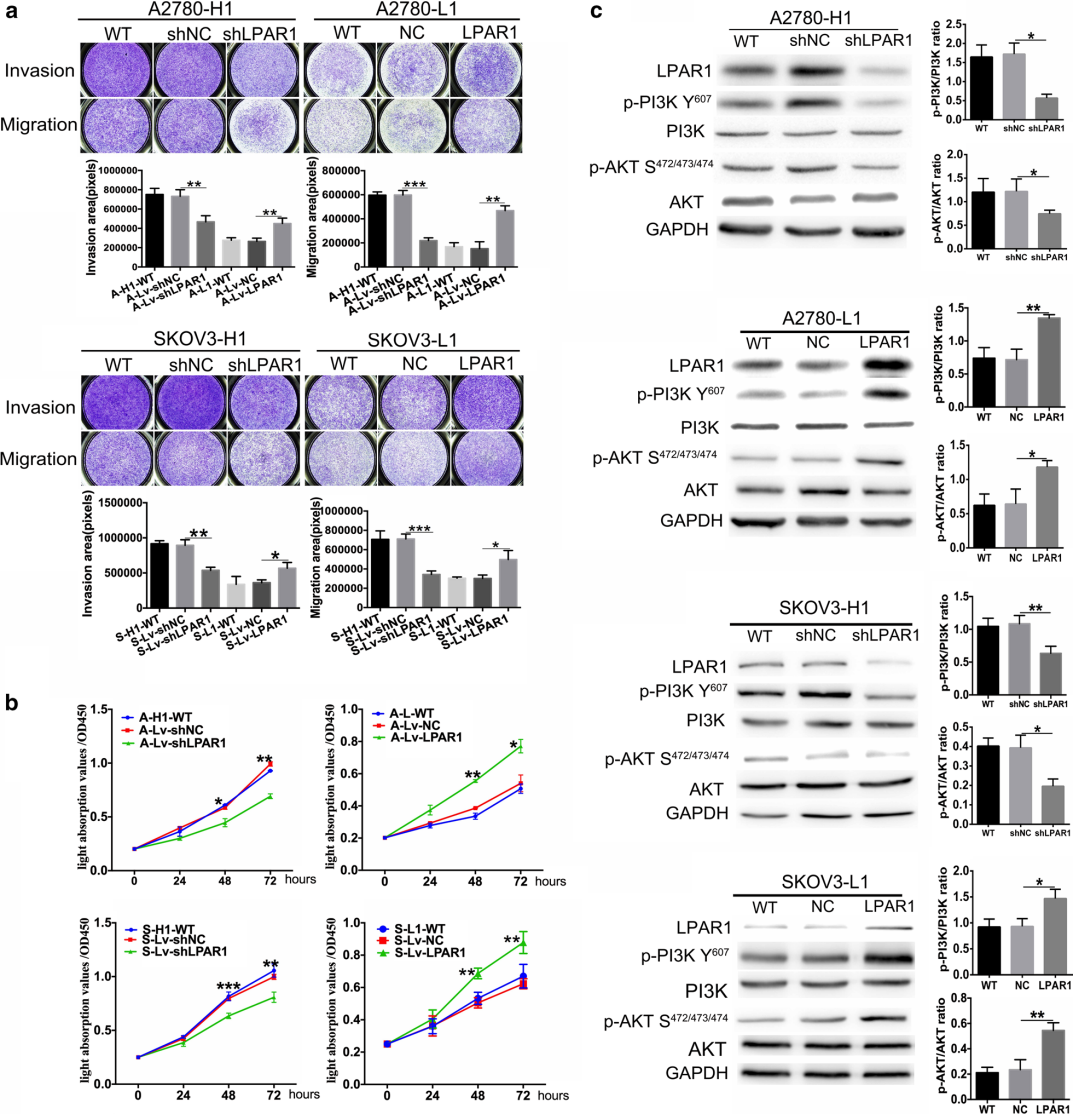
Role of LPAR1 in modulating biological functions and the correlation between LPAR1 expression and the PI3K/AKT pathway. **a** Transwell invasion/migration assays revealed significant decreases in the invasion and migration of the LPAR1 knockdown groups compared with the corresponding control groups (invasion: A2780: 732,395 ± 39,154 vs 467,818 ± 36,623, P = 0.008; SKOV3: 894,327 ± 45,417 vs 537,955 ± 25,284, P = 0.002) (migration: A2780: 598,600 ± 21,514 vs 219,396 ± 13,421, P < 0.001; SKOV3: 711,429 ± 29,264 vs 344,107 ± 20,447, P < 0.001). Similarly, the invasion/migration of the LPAR1-overexpressing cells were significantly increased compared with the corresponding control groups (invasion: A2780: 265,436 ± 19,202 vs 450,506 ± 31,967, P = 0.008, SKOV3: 362,365 ± 22,484 vs 567,782 ± 47,373, P = 0.017) (migration: A2780: 152,081 ± 33,230 vs 468,313 ± 22,950, P = 0.001; SKOV3: 302,498 ± 20,021 vs 497,588 ± 54,052, P = 0.028). **b** A cell proliferation assay was performed using a CCK-8 kit, and the cell growth curves showed that the LPAR1 deficiency inhibited the proliferation of the A-LV-shLPAR1 and S-LV-shLPAR1 cells (48 h: P = 0.040/< 0.001; 72 h: P = 0.005/0.004). In contrast, LPAR1 overexpression accelerated the proliferation of the A-LV-LPAR1 and S-LV-LPAR1 cells (48 h: P = 0.001/0.002; 72 h: P = 0.039/0.004). In addition, differences in the invasion, migration, and proliferation were not observed between the control groups and the corresponding wild-type (WT) groups (all P > 0.05). **c** Western blot analyses revealed significantly decreased levels of PI3K p85 alpha phosphorylated at Y607 (p-PI3K Y^607^) and AKT1/2/3 phosphorylated at S472 + S473 + S474 (p-AKT S^472/473/474^) in the LPAR1 knockdown cells (p-PI3K: P = 0.003/0.009; p-AKT: P = 0.040/0.010) and significantly increased levels in the LPAR1-overexpressing cells (p-PI3K: P = 0.003/0.016; p-AKT: P = 0.017/0.006). In addition, no differences were observed between the control groups and the corresponding wild-type groups (all P > 0.05)

According to the results of the Transwell invasion/migration assays (Fig. 3a), invasion and migration of the LPAR1 knockdown groups were significantly decreased compared with the corresponding control groups (invasion: A2780: 732,395 ± 39,154 vs 467,818 ± 36,623, P = 0.008; SKOV3: 894,327 ± 45,417 vs 537,955 ± 25,284, P = 0.002) (migration: A2780: 598,600 ± 21,514 vs 219,396 ± 13,421, P < 0.001; SKOV3: 711,429 ± 29,264 vs 344,107 ± 20,447, P < 0.001). Similarly, the invasion/migration of the LPAR1-overexpressing groups were significantly increased compared with the corresponding control groups (invasion: A2780: 265,436 ± 19,202 vs 450,506 ± 31,967, P = 0.008, SKOV3: 362,365 ± 22,484 vs 567,782 ± 47,373, P = 0.017) (migration: A2780: 152,081 ± 33,230 vs 468,313 ± 22,950, P = 0.001; SKOV3: 302,498 ± 20,021 vs 497,588 ± 54,052, P = 0.028). A cell proliferation assay was performed using a CCK-8 kit to detect the effect of LPAR1 on cell proliferation (Fig. 3b). Based on the cell growth curves, the LPAR1 deficiency inhibited the proliferation of the A-Lv-shLPAR1 and S-Lv-shLPAR1 cells (48 h: P = 0.040/< 0.001; 72 h: P = 0.005/0.004). In contrast, the LPAR1 overexpression accelerated the proliferation of the A-Lv-LPAR1 and S-Lv-LPAR1 cells (48 h: P = 0.001/0.002; 72 h: P = 0.039/0.004). In addition, differences in invasion, migration, and proliferation were not observed between the control groups and the corresponding wild-type groups (all P > 0.05).

### Role of LPAR1 in tumor formation in vivo

**Fig. 4:**
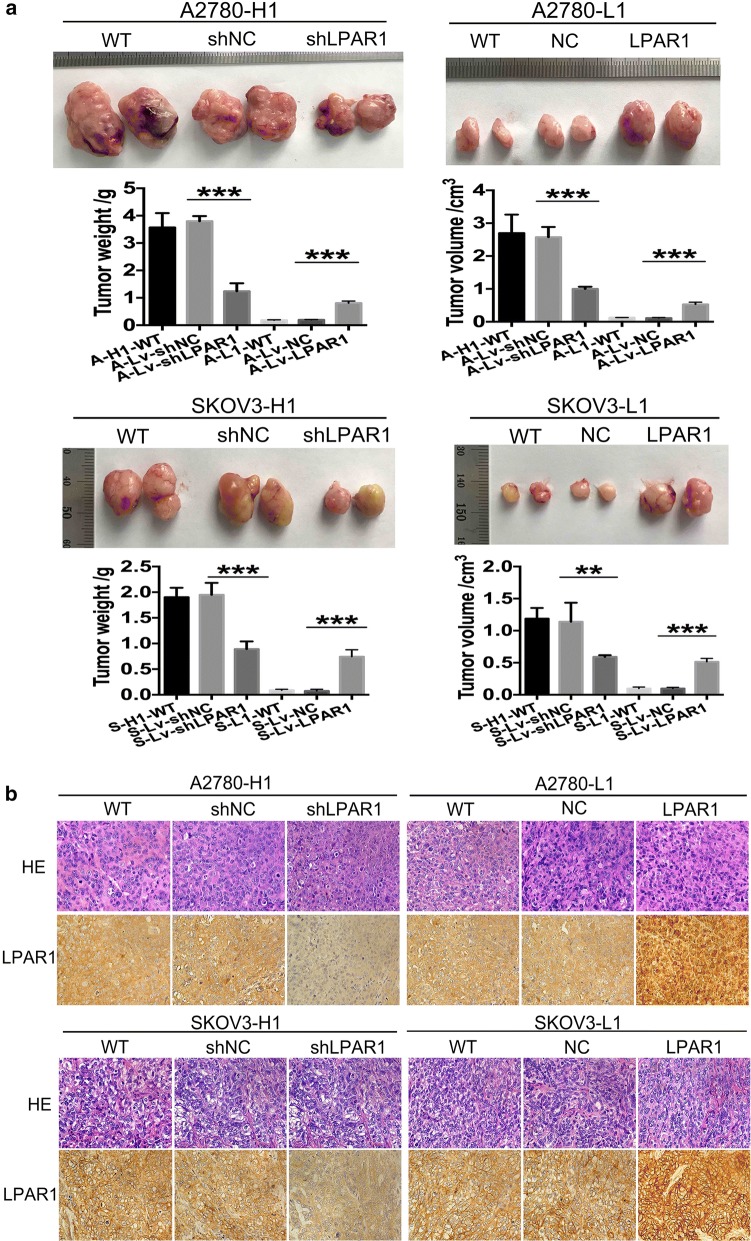
Role of LPAR1 in tumor formation in vivo. Xenograft experiments were performed to test and verify the role of LPAR1 in tumor formation. **a** Compared with the corresponding control groups, the tumors in the A-Lv-shLPAR1 and S-Lv-shLPAR1 groups were significantly smaller in terms of both volume (P < 0.001/= 0.001) and weight (both P < 0.001). In contrast, the tumors in the A-L1-LPAR1 and S-L1-LPAR1 groups were much larger than in the corresponding control groups in terms of both volume (both P < 0.001) and weight (both P < 0.001). In addition, no difference was observed between the control groups and the corresponding wild-type (WT) groups (all P > 0.05). **b** The tumor tissues were fixed formalin, embedded in paraffin, and stained with H&E. IHC were performed to detect LPAR1 expression. LPAR1 expression was detected using IHC, which verified the efficiency of the transduction with the lentiviral particles

Xenograft experiments were performed to test and verify the role of LPAR1 in tumor formation. Twelve groups of nude mice were injected with A-H1-wild-type, A-Lv-shNC, A-Lv-shLPAR1, A-L1-wild-type, A-Lv-NC, A-Lv-LPAR1, S-H1-wild-type, S-Lv-shLPAR1, S-Lv-shNC, S-L1-wild-type, S-Lv-NC, and S-Lv-LPAR1 cells (Fig. 4a). At the end of the observation period (45 days for the A2780 cells and 60 days for the SKOV3 cells), the tumor formation rates in the mice injected with A-L1-wild-type, A-Lv-shNC, S-L1-wild-type and S-Lv-shNC cells were 33.3% (2/6), 50% (3/6), 83.3% (5/6), and 66.7% (4/6), respectively, and the rate in the other groups was 100%. Compared with the corresponding control groups, the A-Lv-shLPAR1 and S-Lv-shLPAR1 groups had tumors with significantly smaller volumes (P < 0.001/= 0.001) and weights (both P < 0.001). In contrast, the tumors in the A-L1-LPAR1 and S-L1-LPAR1 groups were much larger than in the corresponding control groups in terms of both volume (both P < 0.001) and weight (both P < 0.001). In addition, no differences were observed between the control groups and the corresponding wild-type groups (all P > 0.05). The tumor tissues were fixed with formalin, embedded in paraffin, and stained with H&E and IHC to detect LPAR1 expression (Fig. 4b). LPAR1 expression was detected using IHC, which verified the efficiency of the transduction with the lentiviral particles.

### Correlation between LPAR1 expression and the PI3K/AKT pathway

The levels of PI3K p85 alpha phosphorylated at Y607 (p-PI3K Y^607^) and AKT1/2/3 phosphorylated at S472 + S473 + S474 (p-AKT S^472/473/474^) in ITH cell models with stable LPAR1 knockdown and LPAR1 overexpression were detected using Western blotting to explore the correlation between LPAR1 expression and the PI3K/AKT pathway (Fig. 3c). Western blot analyses revealed that significantly decreased levels of p-PI3K and p-AKT in the LPAR1 knockdown cells (p-PI3K: P = 0.003/0.009; p-AKT: P = 0.040/0.010) and significantly increased levels in the LPAR1-overexpressing cells (p-PI3K: P = 0.003/0.016; p-AKT: P = 0.017/0.006) compared with the corresponding control groups. In addition, no differences were observed between the control groups and the corresponding wild-type groups (all P > 0.05).

## Discussion

A heterogeneous mixture of functionally distinct cancer cells exhibiting varying levels of receptor activity and differentiation and distinct metabolic and epigenetic states exists within a tumor [6]. ITH leads to wide range of responses of tumors to therapeutic agents, resulting in many difficulties in clinical treatment. ITH in ovarian cancer, which has been reported in the literature [9, 23] and our previous study [11], is related to tumor metastasis, chemotherapy resistance and recurrence. In the present study, TMAs including four types of matched primary and recurrent tumor lesions obtained from the same patients with OSC were constructed to explore ITH in OSC. In tissues from the same patients, the levels of the LPAR1 protein were noticeably higher in the lymphatic metastatic and recurrent OSC tissues than in the primary tumor lesions. Based on these results, ITH exists in OSC and LPAR1 plays an essential role in the development of ITH. In addition, the LPAR1 protein is expressed at high levels in patients with less than 12 months of PFS or 36 months of OS, as detected by IHC staining. Similarly, Yu et al. [24] reported significantly increased LPAR1 expression in patients with advanced clinical stages of epithelial ovarian cancer, an abdominal metastasis of more than 2 cm, retroperitoneal lymph node metastasis or hepatic metastasis. Based on the previous study and our data, we propose that high expression of LPAR1 represents a potential predictor of a poor prognosis for patients with OSC.

LPAR1 plays as an important role in the development of malignant tumors, including breast cancer, ovarian cancer, and pancreatic cancer, by binding to LPA and activating downstream targets [18, 25, 26]. In cancer cells, LPAR1 contributes to DNA synthesis and cell division following lipid phosphate phosphatase-1 and LPA stimulation [27]. According to Park et al. [28], LPAR1 mediates the LPA-induced migration of ovarian cancer cells. Silencing of LPAR1 alone in HEY and SKOV3 cells significantly reduces LPA-induced invasion [24]. The ovarian cancer stem cell properties induced by LPA stimulation are abrogated by LPAR1-specific inhibitors or LPAR1 silencing [29]. In addition, LPAR1 plays an essential role in the progression of bone metastasis, and antagonists blocking the LPAR1-dependent effects of LPA represent potential therapeutic targets in these patients [30]. Although accumulating evidence have revealed a role for LPAR1 in malignant tumors, few studies have focused on the role of LPAR1 in the development of ITH. In our previous study, we observed significantly increased levels of the LPAR1 protein and mRNA in ITH cell models with high invasive/migratory capacities [11]. Single-cell subclones with distinct invasive/migratory capacities were reisolated, and cell models of ITH were reidentified in this study to explore and verify the role of LPAR1 in the development of ITH. In vitro biological functions, including invasive, migratory, and proliferative capacities, and in vivo tumor formation were significantly decreased in the LPAR1-silenced ITH cell models. In contrast, cellular functions were significantly enhanced in the LPAR1-overexpressing ITH cell models both in vitro and in vivo. Thus, LPAR1 plays a vital role in regulating the viability of independent heterogeneous subsets of OSC cell lines, and the development of ITH in OSC is strongly correlated with LPAR1 expression.

As shown in our previous study, the activation of the PI3K/AKT/mTOR pathway is associated with the development of ITH in human OSC [11]. As a member of the GPCR superfamily, LPAR1 activates the PI3K/AKT pathway by binding to G_αi/o_ proteins [13]. One of the most notable functions of LPAR1-induced activation of G_αi/o_ proteins in rodent Schwann cells is survival signaling via the PI3K/AKT pathway [16]. LPA induces hyaluronic acid synthesis in human skin fibroblasts mainly by activating LPAR1-G_αi/o_, followed by the activation of the PI3K signaling pathway [17]. Sahay et al. [18] reported that LPAR1/PI3K signaling mediates the LPA-dependent metastasis of breast cancer cells. However, to the best of our knowledge, data related to the regulatory effect of LPAR1 on the PI3K/AKT pathway in OSC are lacking. We detected the levels of p-PI3K and p-AKT in LPAR1-silenced and LPAR1-overexpressing ITH cell models to investigate the correlation between LPAR1 expression and the PI3K/AKT pathway and determine the potential mechanisms involved in the development of ITH. The levels of p-PI3K and p-AKT were significantly decreased in the LPAR1 knockdown cells and significantly increased in the LPAR1-overexpressing cells. Based on these results, LPAR1 might be involved in the development of ITH in human OSC by activating the PI3K/AKT signaling pathway. Activation of the PI3K/AKT pathway is involved in carcinogenesis and ovarian cancer development [31, 32]. Certain experts have suggested that PI3K only plays a passive role in the development of human malignancies [33, 34]. The inhibition of PI3K potentially leads to feedback upregulation of the expression and phosphorylation of multiple receptor tyrosine kinases [35]. Researchers have proposed that PI3K inhibitors should be used in combination with other antagonists in cancer therapy [35, 36]. As shown in the present study, the levels of p-PI3K and p-AKT vary according to the expression of the LPAR1 protein. LPAR1, which is located upstream of the PI3K/AKT pathway, may present an effective target for the treatment of ovarian cancer.

Our study provides the first evidence of roles for LPAR1 and the PI3K/AKT pathway in ITH, and a potential treatment target for OSC has been described. However, some important limitations also exist in the present study. First, IHC staining for LPAR1 in human OSC tissues must be interpreted with caution due to the drawbacks of TMAs. TMAs including the limited tissues collected in the present study might not be representative of the whole tumor. In addition, due to the limitations of basic research, the effects of LPAR1 and its correlation with the PI3K/AKT pathway should be verified in the future.

## Conclusions

ITH exists in OSC, as evidenced by heterogeneous LPAR1 expression in primary, lymphatic metastatic and recurrent OSC tissue from the same patients. Higher levels of the LPAR1 protein were associated with a poor prognosis. LPAR1 plays essential roles in the invasion, migration, and proliferation of heterogeneous subsets of OSC cell lines and the development of ITH in OSC, possibly by activating the PI3K/AKT signaling pathway. Strategies that decrease LPAR1 expression represent a potential therapeutic target for OSC.

## Additional files

**Additional file 1: Table S1.** The principle materials and instruments used in the present study.

**Additional file 2: Table S2.** Details of primary antibodies used for Western blotting.

**Additional file 3: Table S3.** The sequences of three specific shRNAs targeting the LPAR1 gene and the scrambled shRNA.

## Data Availability

The datasets used and/or analysed during the current study are available from the corresponding author on reasonable request.

## Abbreviations

ITH: intratumoral heterogeneity
OSC: ovarian serous cystadenocarcinoma
LPAR1: lysophosphatidic acid receptor 1
LPA: lysophosphatidic acid
GPCR: G protein-coupled receptor
FIGO: Federation of Gynecology and Obstetrics
PFS: progression-free survival
OS: overall survival
TMA: tissue microarray
H-score: histochemistry score
FBS: fetal bovine serum
CCK-8: counting Kit-8
eGFP: enhanced green fluorescent protein
qRT-PCR: quantitative real-time polymerase chain reaction
p-PI3K: phosphorylated PI3K
p-AKT: phosphorylated AKT
