# Supplementary material for: LPAR1 regulates the development of intratumoral heterogeneity in ovarian serous cystadenocarcinoma by activating the PI3K/AKT signaling pathway
Source: Cancer Cell Int. 2019 Jul 29;19:201. doi: 10.1186/s12935-019-0920-0 (PMC6664705; doi:10.1186/s12935-019-0920-0)
Supplement: Supplementary file 1 — Additional file 1: Table S1. The principle materials and instruments used in the present study. [file 12935_2019_920_MOESM1_ESM.docx]

| Table S1. The principle materials and instruments used in the present study. | |
| --- | --- |
| Materials/Instruments | Supplier |
| TMA | |
| TMArrayer | UNITMA, Korea |
| TMA block | UNITMA, Korea |
| IHC staining | |
| anti-LPAR1 antibody | Abcom, UK |
| IHC staining evaluation of TMA | |
| Pannoramic MIDI/P250 | 3D HISTECH, Hungary |
| Pannoramic Viewer 1.15.4 software |  |
| Cell culture | |
| RPMI-1640 | HyClone Laboratories, Inc. |
| Fetal bovine serum |  |
| Penicillin and streptomycin |  |
| Dimethyl sulfoxide | Sigma, USA |
| A total of 96-, 48-, 24-, and 6-well plates, as well as culture dishes | Corning Inc, USA |
| Transwell insert invasion/migration assay | |
| Matrigel | BD Biosciences, USA |
| Transwell chambers |  |
| Image Pro Plus software | [Media Cybernetics](http://www.baidu.com/link?url=hEXCf9wtjrcXtMWm7WEQ9e3Mc0HytGsQqxTOfwyqohF5zTCR3QJFjBRopeY2L65m), USA |
| Counting Kit-8 | Dojindo, Japan |
| Western blotting |  |
| Radioimmunoprecipitation assay lysis buffer | Applygen technology, China |
| Bicinchoninic acid | Solabio, China |
| Bio-Rad GelDoc EZ | Hercules, USA |
| Image J software | Rawak Software, Germany |
| Quantitative RT-PCR | |
| TRIzol reagent | Invitrogen, USA |
| NanoDrop ND-2000 spectrophotometer | NanoDrop Technologies, USA |
| PrimeScript RT Reagent Kit with gDNA Eraser | Takara Bio, Japan |
| ABI Prism 7500 RT-PCR system | Thermo Fisher Scientific, USA |
| Production and transduction of lentiviral particles | |
| Lipofectamine 2000 kit | Invitrogen, USA |
| GV248 plasmid | Genechem Co., Ltd, Shanghai, China |
| GV492 plasmid |  |
| Enhanced infection solution |  |
| Polybrene |  |
| Xenograft experiments | |
| Female BALB/c nude mice | Beijing Vital River Laboratory Animal Technology, China |
| Statistical analysis | |
| SPSS 22.0 statistical package | IBM, USA |
| TMA, tissue microarray; IHC, immunohistochemistry. | |
