# Supplementary material for: LPAR1 regulates the development of intratumoral heterogeneity in ovarian serous cystadenocarcinoma by activating the PI3K/AKT signaling pathway
Source: Cancer Cell Int. 2019 Jul 29;19:201. doi: 10.1186/s12935-019-0920-0 (PMC6664705; doi:10.1186/s12935-019-0920-0)
Supplement: Supplementary file 2 — Additional file 2: Table S2. Details of primary antibodies used for Western blotting. [file 12935_2019_920_MOESM2_ESM.docx]

| Table S2. Details of primary antibodies used for Western blotting. | | |
| --- | --- | --- |
| Antibodies | Dilution | Supplier |
| Anti-LPAR1 | 1:1000 | Abcom, UK |
| Anti-PI3K | 1:1000 | Abcom, UK |
| Anti-AKT | 1:1000 | Abcom, UK |
| Anti-PI3K p85 alpha (phospho Y607) | 1:800 | CST, USA |
| Anti-AKT1/2/3 (phospho S472+S473+S474) | 1:500 | Abcom, UK |
| Anti-GAPDH | 1:2000 | Abcom, UK |
