# Supplementary material for: LPAR1 regulates the development of intratumoral heterogeneity in ovarian serous cystadenocarcinoma by activating the PI3K/AKT signaling pathway
Source: Cancer Cell Int. 2019 Jul 29;19:201. doi: 10.1186/s12935-019-0920-0 (PMC6664705; doi:10.1186/s12935-019-0920-0)
Supplement: Supplementary file 3 — Additional file 3: Table S3. The sequences of three specific shRNAs targeting the LPAR1 gene and the scrambled shRNA. [file 12935_2019_920_MOESM3_ESM.docx]

| Table S3. The sequences of three specific shRNAs targeting the LPAR1 gene and the scrambled shRNA | |
| --- | --- |
| shRNA | Sequence |
| LPAR1-shRNA-1* | CTATGAGAAATTCTTCCTT |
| LPAR1-shRNA-2 | GGTCATGGTGGCAATCTATGT |
| LPAR1-shRNA-3 | GCAATCGAGAGGCACATTACG |
| nonspecific scrambled shRNA | TTCTCCGAACGTGTCACGT |
| shRNA: short hairpin RNA; LPAR1: lysophosphatidic acid receptor 1. *: The most effective shRNA sequence selected based on the results of Western blotting and qRT-PCR in the preliminary experiment. | |
